# End-of-life decisions and practices for very preterm infants in the Wallonia-Brussels Federation of Belgium

**DOI:** 10.1186/s12887-018-1168-x

**Published:** 2018-06-26

**Authors:** Isabelle Aujoulat, Séverine Henrard, Anne Charon, Anne-Britt Johansson, Jean-Paul Langhendries, Anne Mostaert, Danièle Vermeylen, Gaston Verellen, on behalf of the 11 neonatal intensive care units in the Wallonia-Brussels Federation, Masendu Kalenga, Isabelle Broux, Pierre Maton, Jean-Paul Langhendries, Elisabeth Henrion, Anne Mostaert, Anneliese Dussart, Marie-Françoise Müller, Eric Cavatorta, Yoann Maréchal, Serge Vanden Eijnden, Chantal Lecart, Anne Charon, Dominique Haumont, Inge Van Herreweghe, Anne-Britt Johansson, Vinciane Vlieghe, Bart Van Overmeire, Danièle Vermeylen, Christian Debauche, Marc Flausch, Brigitte Sepulchre

**Affiliations:** 1 Université catholique de Louvain (UCL), Institute of Health and Society (IRSS), Clos chapelle-aux-champs, n° 30.14 - 1200, Brussels, Belgium; 2 Grand Hôpital de Charleroi (GHC), Charleroi, Belgium; 3 Hôpital universitaire des enfants Reine Fabiola (HUDERF), Brussels, Belgium; 4 CHC-Clinique Saint-Vincent, Rocourt, Belgium; 5 Centre hospitalier régional (CHR) de Namur, Namur, Belgium; 6 Hôpital Erasme, Brussels, Belgium; 7 Cliniques universitaires Saint-Luc, Brussels, Belgium

**Keywords:** Preterm birth, End-of-life, NICU, Survey, Belgium

## Abstract

**Background:**

Very preterm birth (24 to < 32 week’s gestation) is a major public health issue due to its prevalence, the clinical and ethical questions it raises and the associated costs. It raises two major clinical and ethical dilemma: (i) during the perinatal period, whether or not to actively manage a baby born very prematurely and (ii) during the postnatal period, whether or not to continue a curative treatment plan initiated at birth. The Wallonia-Brussels Federation in Belgium counts 11 neonatal intensive care units.

**Methods:**

An inventory of key practices was compiled on the basis of an online questionnaire that was sent to the 65 neonatologists working in these units. The questionnaire investigated care-related decisions and practices during the antenatal, perinatal and postnatal periods, as well as personal opinions on the possibility of standardising and/or legislating for end-of-life decisions and practices. The participation rate was 89% (*n* = 58).

**Results:**

The results show a high level of homogeneity pointing to overall agreement on the main principles governing curative practice and the gestational age that can be actively managed given the current state of knowledge. There was, however, greater diversity regarding principles governing the transition to end-of-life care, as well as opinions about the need for a common protocol or law to govern such practices.

**Conclusion:**

Our results reflect the uncertainty inherent in the complex and diverse situations that are encountered in this extreme area of clinical practice, and call for qualitative research and expert debates to further document and make recommendations for best practices regarding several “gray zones” of end-of-life care in neonatology, so that high quality palliative care may be granted to all neonates concerned with end-of-life decisions.

**Electronic supplementary material:**

The online version of this article (10.1186/s12887-018-1168-x) contains supplementary material, which is available to authorized users.

## Background

Premature birth is a major public health issue due to its prevalence, the clinical and ethical questions it raises and the associated costs. In Belgium, an important process of collective reflection was initiated several years ago through the EPIBEL study, which retrospectively reviewed the case files of 525 babies born at gestational ages of between 22 and 26 weeks in 19 perinatal care units between 1 January 1999 and 1 January 2001 [1, 2]. Of the 303 babies who were born alive and admitted to a neonatal intensive care unit (NICU), 128 (40%) had died during their stay in the unit and 175 had survived [2]. A clinical examination carried out at 3 years of age for 89 of the children who had survived showed a survival rate with no physical, neurological or cognitive sequelae of approximately 40% and a survival rate with sequelae of 60%, of whom 28% had a major disability [1]. The observation of frequent, severe long-term morbidity in children who have survived a birth at the threshold of viability concurs with findings from other European studies [3–5]. It raises two major clinical and ethical questions, which come up again and again: (i) during the perinatal period, whether or not to actively manage a baby born very prematurely, and (ii) during the postnatal period, whether or not to continue a curative treatment plan initiated at birth.

The results of a number of surveys carried out internationally regarding self-reported practices of neonatologists have highlighted a range of opinions and practices concerning which therapeutic approach to adopt, especially during the period of uncertainty between 23 and 25 weeks’ gestation [6–8]. In view of the uncertain outcomes for these infants and the dilemma involved in maximising their chances of survival while minimising both the impact on future quality of life and any avoidable suffering during the perinatal period, two extreme positions appear to be dominant among healthcare teams: the first is to systematically undertake standby resuscitation to give all babies a chance of survival. The findings of a prospective national study, carried out in Sweden in a cohort of 507 babies who were followed up for two and a half years, appear to support this approach, provided that care is given in highly specialised centres [9]. The results of the Swedish study actually show an increase in survival rates with no increase in the risk of neurodevelopmental sequelae in units practising systematic active management of extremely premature babies, including those between 22 and 24 weeks’ gestation [9]. The second approach is to refer systematically to a protocol in order to make decisions on the basis of a number of predefined, objective criteria (such as gestational age and weight, based on the results of epidemiological studies and/or experience within a department). This option has been adopted in the Netherlands, where the “Groningen Protocol” was developed in 2004 [10]. In France, without going as far as establishing a formal protocol on required practice, a think-tank on ethical aspects in perinatal care has put forward a number of clinical guidelines aiming at trying to reconcile the legal requirements and ethical responsibility [11, 12]. These guidelines stress the importance of the deliberative processes involved in decision making, where decisions to actively manage a baby are based on an individualised assessment of each situation together with colleagues and the parents [13, 14].

In Belgium, the process of reflection that was initiated at national level in the context of the EPIBEL study [1, 2] is continuing in the form of differentiated regional initiatives. In the Flemish Region, since 2014, care-related decisions and practices in situations of extreme prematurity have been set out in a consensus document, which has the status of a protocol [15]. Such a protocol does not currently exist in the Wallonia-Brussels Federation, which has 11 NICUs. In 2013, these NICUs wished to come together to set up a common research project with the aim of working collectively to think through and optimise practices in relation to curative treatment and/or palliative care provided to extremely preterm infants, paying special attention to babies born at the threshold of viability (between 23 and 26 weeks). The purpose of this study was to highlight the similarities and possible areas of variability between the neonatologists at the 11 NIC units in relation to: (i) their attitudes and experiences with regard to care-related decisions and practices in situations of extreme prematurity and (ii) their opinions and wishes relating to standardising and/or legislating for care-related decisions and practices in situations of extreme prematurity.

## Methods

### Study design and data collection

An online survey, based on self-administered questionnaires, was conducted among all neonatologists working in the 11 NIC units in the Wallonia-Brussels Federation of Belgium in 2014. Each of the 65 neonatologists who worked in these units at the time of the survey received a personal invitation to complete an anonymous online questionnaire which addressed: (i) care-related decisions and practices during the antenatal, perinatal and postnatal periods for very preterm infants; (ii) the neonatologists’ personal opinions on the possibility of standardising and/or legislating for care-related decisions and practices in intensive neonatology.

A first draft of the questionnaire had been elaborated by the members of a steering committee (authors 3 to 8), based on their knowledge of the literature and the questions predominantly raised in their own clinical practice. This steering committee had been nominated by the 11 neonatal intensive care units, and received methodological advice from the first and second authors. The online questionnaire was hosted on limesurvey and pilot-tested by the first author among 3 neonatologists from 3 different neonatal care units. Sample questions relating to the various areas of investigation are presented in Table 1.

**Table 1:** Sample questions (full questionnaire upon request) Additional file 1

Areas of investigation	Sample questions
Decisions and practices in antenatal period	- *Where applicable, who is involved in the decision-making process regarding advanced decisions to actively manage or not an extremely premature baby at birth?* - *In your centre, at which gestational age may a very premature baby be actively managed?*
Decisions and practices in perinatal period	- *Which anamnestic criteria are considered when making the decision to actively manage an extremely premature baby? Where applicable, are the parents invited to send a formal consent form to withhold or withdraw treatment?*
Decisions and practices in postnatal period	- *Do you have a written protocol or a standardised procedure to ensure the baby’s comfort in the context of palliative care?* - *In the context of a palliative care pathway, do you ever practise “active” end-of-life (use of analgesic and/or sedative drugs at above therapeutic doses)?*
Opinions regarding standardization of and legislation on end-of-life care	- *In making end-of-life decisions, have you ever experienced fear of litigation?* (possible responses: Yes, I have; Yes, it could happen; No; I don’t know) - *Would you like “active” end-of-life practices in situations of extreme prematurity* to be ALLOWED *by a protocol or a law?* (possible responses: Yes; No; Uncertain)

### Participants

Of the 65 eligible neonatologists, 58 (89.2%) responded to the questionnaire, including 40 women (69.0%) and 18 men (31.0%). Table 2 shows the breakdown of the respondents according to their age and the number of years of experience in neonatology.

**Table 2:** Breakdown of respondents by age group and number of years of experience in neonatology

Years of experience Age	5 years or less n (%)	6–14 years n (%)	15 years or more n (%)	TOTAL n (%)
Under 35	7 (12.1)	0 (0.0)	0 (0.0)	7 (12.1)
35–55	5 (8.6)	24 (41.4)	8 (13.8)	37 (63.8)
Over 55	0 (0.0)	1 (1.7)	13 (22.4)	14 (24.1)
TOTAL	12 (20.7)	25 (43.1)	21 (36.2)	58 (100.0)

### Data analysis

Categorical variables are presented using numbers and percentages. All categorical variables were compared using Pearson’s chi-squared test, chi-squared test using Yate’s correction for continuity, Fisher’s exact test or Fisher-Freeman-Halton exact test, as appropriate. The proportions of responses to the various questions were compared for neonatologists of both genders (male versus female), based on the age of the neonatologist (under 35, 35–54 or over 55) and based on the number of years of experience in neonatology (5 years or less, 6–14 years, or 15 years or more). All statistical analyses were performed using R software Version 3.2.1 (Free software Foundation Inc., Boston, MA). A *p*-value < 0.05 was considered to be statistically significant.

In addition to the general report, which set out the results for the 11 units, each neonatologist who was invited to participate in the study (*n* = 65) received an appendix to the general report, containing the results for his or her unit only. The results for each NICU were considered to be confidential and were communicated by the research team only to the units in question, so that within each team, comparisons could be made with the pooled results of the 11 NICUs. The results were first discussed separately within each team, and later discussed and commented in the presence of the researchers, in order to acknowledge common reported practices and discuss the reasons behind diverse opinions or reported practices. The results hereafter are presented according to (i) what has been validated by the 11 centres as shared good practices; (ii) what has been acknowledged as divergent to some extent, with possible areas for standardisation and improvement.

### Ethical considerations

Our study did not involve any patients nor patients’ relatives, nor did it require that patient data be shared with the researchers. Our research therefore does not fall within the scope of the Belgian Law of 7 May 2004 on Human Experiments, and did therefore not require the approval of an ethics committee, nor that informed consent be signed by the participants. However, the representatives of the 11 participating neonatal care units individually informed their respective ethics committees of the study.

## Results

### Overall agreement on the main principles governing the gestational age that can be managed and the role of parents in making decisions to actively manage or not a baby

#### Antenatal decisions

The respondents unanimously stated (100%, *n* = 58/58) that they do make anticipated decisions in the antenatal period, concerning whether or not to resuscitate at birth. Yet, at the time of birth, an antenatal decision may be revised on the basis of clinical assessment of the baby’s maturity, its vitality at birth and the presence of malformations not detected during pregnancy. The parents are most always involved in the deliberative process regarding antenatal decisions to resuscitate or not a baby (96.5%, *n* = 56/58). Before 26 weeks, their influence is almost as significant as that of the neonatologist. After 26 weeks, their opinion has less of an influence on the decision, and the role of the neonatologist becomes dominant, as shown in Table 3.

**Table 3:** Parental and medical influence regarding antenatal decisions before and after 26 weeks of gestational age

	≤ 26 weeks		26 weeks	
	Parents n (%)	Neonatologists n (%)	Parents n (%)	Neonatologists n (%)
1 – Most influential	24 (42.9)	25 (44.6)	9 (16.4)	44 (78.6)
2 – Very Influential	17 (30.4)	18 (32.1)	21 (38.2)	5 (8.9)
3 – Somewhat influential	12 (21.4)	8 (14.3)	17 (30.9)	2 (3.6)
4 – Less influential	3 (5.4)	5 (8.9)	8 (14.5)	5 (8.9)
*Missing values*	*2 (3.4)*	*2 (3.4)*	*3 (5.2)*	*2 (3.4)*

#### Perinatal decisions in cases of emergency delivery

Where no decision was made beforehand, standby resuscitation is always possible and gives time to clarify the situation. At around 24 weeks, the decision on whether or not to resuscitate a child born at the threshold of viability becomes acute. At less than 24 weeks, neonatology teams rarely (3.5%, *n* = 2/57, 1 missing value) agree to initiate resuscitation for an infant. After 24 weeks, babies are almost always (96.5%, *n* = 55/57, 1 missing value) managed intensively and maturation with corticosteroids begins at between 23 and 24 weeks (96.4%%, *n* = 55/57, 1 missing value) (data not shown).

#### Medical and psychosocial criteria considered for decision

Although the respondents agreed that their decisions are never based on single criteria, there was a rather strong consensus in the responses concerning medical criteria that are involved in decisions on whether or not to resuscitate, as shown in Table 4. The criteria that are taken into account in the majority of cases are the presence of a significant malformation, chorioamnionitis or other infection, birth weight and signs of acute foetal distress. The criteria that are not taken into account in the majority of cases are the phenotype and sex of the baby. As far as possible psychosocial criteria are concerned, such as for instance the parents’ socioeconomic background, the mother’s age or drug addiction, these were considered only on an individual basis and their importance may not be generalised.

**Table 4:** Medical criteria always or often considered in antenatal and postnatal decisions to actively manage or not an extremely premature baby

	Antenatal decisions n (%)	Postnatal decisions n (%)
Presence of a significant malformation	52 (92.9) 2 missing values	50 (87.7) 1 missing value
Chorioamnionitis or other infection	44 (77.2) 1 missing value	43 (75.4) 1 missing value
Birth weight	43 (75.5) 1 missing value	37 (64.9) 1 missing value
Signs of acute foetal distress	32 (57.1) 2 missing values	41 (71.9) 1 missing value
Singleton/multiple	16 (29.1) 3 missing values	9 (16.4) 3 missing values
Phenotype	4 (7.1) 2 missing values	1 (1.8) 2 missing values
Sex	6 (10.7) 2 missing values	3 (5.4) 2 missing values

#### Participation of parents in decisions to initiate or withhold curative treatment

At the time of birth, the parents are involved in the decision to actively manage the baby or not, but to a lesser extent than when decisions are made beforehand during the antenatal period. Whereas the neonatologist is always involved according to 94.7% (*n* = 54/57, 1 missing value) of respondents, the parents are always involved according to 39.3% (*n* = 22/56, 2 missing values) of respondents, and are often or sometimes involved according to 53.5% (*n* = 30/56, 2 missing values) of respondents. The numerous comments received in relation to this issue all stress the importance of the deliberative process, meant to reach an agreement that is most satisfying for both the parents and the healthcare team. In fact, all respondents (100%, *n* = 53/53, 5 missing values) reported that a stand-by resuscitation is always possible, in order to allow sufficient time to reach the best decision. When a decision is made to not actively manage a baby, the parents are only exceptionally invited to sign an informed consent form: Indeed, parents are never invited to sign such a form according to 91.2% (*n* = 52/57, 1 missing value) of the respondents.

#### Postnatal participation of parents in decisions to continue or withdraw curative treatment

As shown in Table 5, where applicable in the postnatal period, the decision to stop curative treatment and provide palliative care always involves the neonatologist (100% of the respondents, *n* = 58/58), and almost always the parents (90.9%, *n* = 50/55, 3 missing values). Again, the responsibility for the final decision mainly lies in the hands of the neonatologists, and not in that of the parents. The nurse, the psychologist and possibly another specialist doctor are often involved in such decisions. External advice, for instance from an ethics committee, is rarely sought (data not shown). The decision to stop curative treatment, where applicable, is mostly influenced by the baby’s expected subsequent quality of life, the subsequent prognosis concerning morbidity and the short or medium term prognosis of survival (Table 6). Although it is common practice to involve the parents in such decisions, a minority of the respondents reported that in exceptional cases they might initiate a palliative care pathway without informing the parents (response “yes” or “uncertain” by 5 respondents/54, 9,3%, 4 missing values) or against the parents’ wish (response “yes” or “uncertain” by 9 respondents/55, 16,3%, 3 missing values).

**Table 5:** Role of parents and neonatologists in decision to continue or withdraw curative treatment in postnatal period

	Involved in deliberative process of decision-making		In charge of making the final decision (outcome)			
			GA ≤ 26 week		GA ≥ 26 week	
	Parents n (%)	Neonatologist n (%)	Parents n (%)	Neonatologist n (%)	Parents n (%)	Neonatologist n (%)
Always	40 (72.7)	56 (100.0)	7 (13.5)	51 (91.1)	10 (19.6)	51 (92.7)
Often	10 (18.2)	0 (0.0)	13 (25.0)	5 (8.9)	9 (17.6)	4 (7.3)
Sometimes	4 (7.3)	0 (0.0)	12 (23.1)	0 (0.0)	10 (19.6)	0 (0.0)
Never	1 (1.8)	0 (0.0)	20 (38.5)	0 (0.0)	21 (41.2)	0 (0.0)
Don’t know	0 (0.0)	0 (0.0)	0 (0.0)	0 (0.0)	1 (2.0)	0 (0.0)
*Missing values*	*3 (5.2)*	*2 (3.4)*	*6 (10.3)*	*2 (3.4)*	*7 (12.1)*	*3 (5.2)*

**Table 6:** Criteria influencing the decision to withdraw curative treatment

	The baby’s expected subsequent quality of life n (%)	The family’s expected subsequent quality of life n (%)	The short or medium term prognosis of survival n (%)	The subsequent prognosis concerning morbidity n (%)	The parents’ subjective experience at the time of birth n (%)
Always	42 (75.0)	8 (14.5)	35 (62.5)	37 (66.1)	5 (9.3)
Almost always	11 (19.6)	15 (27.3)	11 (19.6)	10 (17.9)	3 (5.6)
Often	2 (3.6)	17 (30.9)	8 (14.3)	5 (8.9)	10 (18.5)
Sometimes	1 (1.8)	12 (21.8)	2 (3.6)	4 (7.1)	16 (29.6)
Rarely	0 (0.0)	1 (1.8)	0 (0)	0 (0.0)	9 (16.7)
Never	0 (0.0)	2 (3.6)	0 (0)	0 (0.0)	11 (20.4)
*Missing values*	*2 (3.4)*	*3 (5.2)*	*2 (3.4)*	*2 (3.4)*	*4 (6.9)*

### Some diversity in opinions and practices regarding the transition to and performance of end-of-life care

#### Standardisation of end-of-life care

At the time of birth or during the postnatal period, where a decision is made not to initiate or continue active management of the baby, the neonatologists were asked whether there is a written protocol or standardised procedure within their unit to ensure that the baby remains comfortable in the context of palliative care. The responses to this question were divided, with almost half of the respondents (47.3%, *n* = 26/55, 3 missing values) answering “no”, while a further 47.3% (*n* = 26/55, 3 missing values) answered “yes”. As shown in Table 7, The fact that they were aware or unaware of a protocol within their unit differed significantly with the respondents’ number of years of experience in neonatology (*p* = 0.014). In fact, 75% of neonatologists with ≤5 years’ experience in neonatology stated that they were not aware of the existence of such a protocol, as compared with 39% of neonatologists with 6 to 14 years’ experience and 40.0% of neonatologists with at least 15 years’ experience. As regards possible variability between units, 100% of the respondents in three of the eleven units stated that a protocol did exist within their own unit. The responses in the other eight units were variable, which again seems to suggest that if a protocol does exist, not everyone knows about it. When a palliative care protocol does exist or is known to the respondents (*n* = 26/55; 3 missing values, 47.3%), it is solely drug-related according to 42% of the respondents (*n* = 11/26), non-drug related (e.g. comfort care with skin-to-skin contact, swaddling, presence of the parents, etc.) according to 23% of the respondents (*n* = 6/26) and mixed (drug-related + other) according to 35% of the respondents (*n* = 9/26). Whether in addition to or in the absence of formal protocols, we should note that the neonatologists stated that individualised palliative care pathways are almost systematically designed and formalised in cases where curative treatment is withdrawn. These individualised care pathways evolve continuously and are reassessed as the situation develops.

**Table 7:** Written protocols or standardised procedures to ensure the baby’s comfort in the context of palliative care? Breakdown of responses according to the neonatologist’s years of experience in neonatology

		Neonatologist’s years of experience in neonatology		
	Total	5 years or less	6–14 years	15 years or more
Response to the question	n (%)	n (%)	n (%)	n (%)
No protocol	26 (47.3)	9 (75.0)	9 (39.1)	8 (40.0)
Drug-related protocol	11 (20.0)	1 (8.3)	8 (34.8)	2 (10.0)
Other protocol	6 (10.9)	0 (0.0)	3 (13.0)	3 (15.0)
Drug-related and other protocol	9 (16.4)	0 (0.0)	2 (8.7)	7 (35.0)
Don’t know	3 (5.5)	2 (16.7)	1 (4.3)	0 (0.0)
*Missing values*	*3 (5.2)*	*0 (0.0)*	*2 (8.0)*	*1 (4.8)*

Fisher-Freeman-Halton exact test: *p*-value = 0.014

#### “Active” end-of- life practices

As shown in Table 8, a large majority of the respondents (76.9%, *n* = 40/52, 6 missing values) stated that they might perform «active» end-of-life practices in the context of a palliative care pathway. Nevertheless, 21.2% (*n* = 11/52, 6 missing values) never would do so and one person responded “I don’t know”. The proportion of responses to this question differs significantly depending on the number of years of experience in neonatology (*p* = 0.007; Table 8). In fact, 62.5% (*n* = 5/8, 4 missing values) of neonatologists with ≤5 years’ experience stated that they would never practise «active» end of life in the context of a palliative care pathway, as compared with 21.8% (*n* = 5/24, 1 missing value) of neonatologists with 6 to 14 years’ experience and 5% (*n* = 1/20, 1 missing value) of neonatologists with at least 15 years’ experience. The most common reasons for the decision to practise an «active» end-of-life are to alleviate unbearable suffering on the part of the baby (91.1%, *n* = 41/45, 13 missing values) or to avoid a poor future quality of life due to a major disability (84,1%, *n* = 37/44, 14 missing values). Rarely, such a decision may be made under the influence of parental pressure or pressure from the healthcare team (see Table 9).

**Table 8:** Experience of “active” end-of-life practices in the context of a palliative care pathway (use of analgesic and/or sedative drugs at above therapeutic doses)? Breakdown of responses according to the neonatologist’s number of years of experience in neonatology

		Neonatologist’s years of experience		
	Total	5 years or less	6–14 years	15 years or more
Response to the question	n (%)	n (%)	n (%)	n (%)
Yes	40 (76.9)	3 (37.5)	18 (75.0)	19 (95.0)
No	11 (21.2)	5 (62.5)	5 (20.8)	1 (5.0)
Uncertain	1 (1.9)	0 (0.0)	1 (4.2)	0 (0.0)
*Missing values*	*6 (10.3)*	*4 (33.3)*	*1 (4)*	*1 (4.8)*

Fisher-Freeman-Halton exact test: *p*-value = 0.007

**Table 9:** Reasons for “active” end-of-life practices

	Avoid poor quality of life due to major disability n (%)	Faced with unbearable suffering of the baby n (%)	Faced with parental pressure n (%)	Faced with pressure from colleagues n (%)
Always	28 (63.6)	29 (64.4)	0 (0.0)	0 (0.0)
Often	9 (20.5)	12 (26.7)	2 (4.5)	2 (4.7)
Sometimes	4 (9.1)	0 (0.0)	11 (25.0)	11 (25.6)
Rarely	0 (0.0)	3 (6.7)	14 (31.8)	5 (11.6)
Never	2 (4.5)	0 (0.0)	16 (36.4)	24 (55.8)
Don’t know	1 (2.3)	1 (2.2)	1 (2.3)	1 (2.3)
*Missing values*	*14 (24.1)*	*13 (22.4)*	*14 (24.1)*	*15 (25.9)*

#### Uncertainty regarding whether “active” end-of-life practices should be allowed and standardised

*“A*ctive” end-of-life practices are currently prohibited in Belgium. When asked if they were in favour of such practices to be allowed and standardised, half of the respondents said yes, with a preference for a protocol rather than a law (see Table 10). In fact, 25.5% (*n* = 13/57, 7 missing values) of them stated that they do not wish to have a law, as compared with only 10% (*n* = 5/50, 8 missing values) who do not wish to have a protocol. Moreover, a large proportion of the respondents appeared to feel “uncertain” about whether or not they wished to have a protocol (38%, *n* = 19/50, 8 missing values) or a law (27.5%, *n* = 14/51, 7 missing values) to govern “active” end-of-life practices.

**Table 10:** Opinions regarding standardisation of or legislation on « active » end-of-life practices?

	In favour of standardisation of practices in a protocole n (%)	In favour of a legal framework to authorise practices n (%)
Yes	26 (52.0)	24 (47.1)
No	5 (10.0)	13 (25.5)
Uncertain	19 (38.0)	14 (27.5)
*Missing values*	*8 (13.8)*	*7 (12.1)*

As shown in Table 11, the proportion of the various responses to the question on whether or not «active» end-of-life practices should be legalised differs significantly depending on the age of the neonatologist (*p* = 0.012) and the number of years spent working in neonatology (*p* = 0.013). Thus 80% (*n* = 4/5, 2 missing values) of neonatologists under 35 years of age were in favour of a law, as compared with 55.9% (*n* = 19/34, 3 missing values) of neonatologists aged 35 to 55, and 8.3% (*n* = 1/12, 2 missing values) of neonatologists over 55 years of age. Moreover, 77.8% (*n* = 7/9, 3 missing values) of neonatologists with 5 years’ experience or less were in favour of a law, as compared with 58.3% (*n* = 14/24, 1 missing value) of neonatologists with 6 to 14 years’ experience and 16.7% (*n* = 3/18, 3 missing values) of neonatologists with at least 15 years’ experience.

**Table 11:** Perceived need for a law to authorise “active” end-of-life practices. Breakdown of responses according to the neonatologists’ age and professional experience

	Age*			Number of years in neonatology**		
	< 35 yrs	35–55 yrs	> 55 yrs	< 5 yrs.	5–14 yrs	≥ 15 yrs
	n (%)	n (%)	n (%)	n (%)	n (%)	n (%)
Yes	4 (80.0)	19 (55.9)	1 (8.3)	7 (77.8)	14 (58.3)	3 (16.7)
No	1 (20.0)	7 (20.6)	5 (41.7)	1 (11.1)	6 (25)	6 (33.3)
Uncertain	0 (0.0)	8 (23.5)	6 (50.0)	1 (11.1)	4 (16.7)	9 (50)
*Missing values*	*2 (28.6)*	*3 (8.1)*	*2 (14.3)*	*3 (25)*	*1 (4)*	*3 (14.3)*

* Fisher-Freeman-Halton exact test: *p*-value = 0.012

** Fisher-Freeman-Halton exact test: *p*-value = 0.013

During the discussions that followed the presentation of our results, the representatives of the 11 neonatal intensive care units stated that most of them did not want decisions and practices concerning «active» end-of-life in situations of extreme prematurity to be regulated by a restrictive law. Most of them, however, supported developing a protocol that would define a framework to guide teams in their decisions and practices, while leaving plenty of scope for individualised care. In fact, most of the comments that were received in connection with this question on whether or not people want standardisation or legislation concerning practice, tended to err on the side of caution in regard to possible standardisation/legislation:

*“In neonatology, the situation of every child at the start of their life is unique and specific... it cannot be set in stone in a protocol, let alone a law.”*

*“Sometimes it is difficult to make a decision as things stand, since the law is completely vague about births at between 24 and 26 weeks. Even if an appropriate legal framework were in place, however, these decisions would still be difficult to make due to the uncertainty over the future development of these children.”*

*“I would like a protocol to set out some markers that are useful for the team and for families while leaving plenty of room for individualisation depending on the situation in each case.”*

## Discussion

The main strengths of our study lie in the collaborative bottom-up approach which involved all 11 NIC units of the French speaking community of Belgium in a collective (across units) and individual (within units) reflective process regarding their current practices, with a chronology from antenatal to postnatal care. The response rate of 89% shows the relevance of such an approach. However, given the small sample size (*N* = 58), the results of the statistical tests should be interpreted with caution. Other limits of our study are inherent to our choice to use a self-administered questionnaire, which does not allow for an individual understanding of complex and dynamic situations. This limit was somewhat counterbalanced by the process of organizing discussions to reflect on the results within and across the different units. Another limit is related to our choice to limit our study to the neonatologists. The opinions and attitudes of the multidisciplinary healthcare teams and the obstetricians from the maternal intensive care units were therefore not sought. However, we are well aware that care practices at the threshold of viability, such as the very early use of antenatal corticosteroids or magnesium treatment, require important collaborative work with the obstetrical team for the best possible outcomes for mother and child. This perinatal collaboration is indeed ongoing amongst the different NIC units (all linked to Maternal Intensice Care units), although it was not documented in this study.

The results of this survey show a high level of homogeneity and general agreement on principles governing the decisions to initiate (versus withhold) and continue (versus (withdraw) curative treatment, that reflect the current state of knowledge, and are congruent with published guidelines and protocols around the world [15, 16], including the consensus guidelines issued in the Flemish Region of Belgium [17], which recommend individualised decision for care for infants born at 23 to 24 weeks’ gestation.

The results of the survey and the subsequent discussions held within and between the different NICUs also demonstrate that the different teams share a same vision regarding the extent of the parents’ role and responsibility regarding possible end-of-life decisions. Again, this vision is congruent with agreed international recommendations on how to communicate with parents around these extremely complex and painful issues [16]. Whether in the antenatal, perinatal or postnatal period, parents are partners in the decision-making process but are not held responsible for the final decision. In practice, whenever it is possible (in rare cases, the father may be absent and the mother in intensive care and therefore not able to participate), the parents’ feelings, wishes and fears are explored and discussed. Moreover, their opinion and preferences are sought, in a deliberative process which involves the various members of the healthcare team -and only marginally some external advisors, such as the members of an ethics committee or a mobile palliative care unit. In any case, stand-by resuscitation may be performed, in order to allow for sufficient time to reach a consensus for the best possible decision to be taken. However, when the final decision is reached, the responsibility for it lies within the hands of the medical team, not within the hands of the parents, who are usually not invited to sign a formal consent form. This important principle, which fully acknowledges and respects the parents’ vulnerability at a time when their child’s birth and death are nearly concomitant, is shared across the different NICU teams. This principle is congruent with the logic of care, where autonomy goes beyond individual choice and self-determination, but is understood as embedded in meaningful relationships where important decisions in situations of vulnerability and uncertainty may be displaced and deferred to well-trusted experts [18, 19].

Going back to the results of our study, some diversity was found in responses regarding the transition to and provision of palliative care, as well as diversity regarding “active” end-of-life practices, and whether they should be allowed through a protocol or a law. The differences, even though these are not always statistically significant, were dependent on age and number of years of professional experience in neonatology. In fact, it is likely that the newest doctors had not yet faced all the situations mentioned in the questionnaire. As far as palliative care practices are concerned, only half of the respondents were aware of a protocol or standardised procedure within their NICU, and the responses also indicate that differences exist between centres in how palliative care is defined (whether drug related, non-drug related or both). During the discussions around the results of the study, it was stressed however that consistency can exist without a written protocol, particularly in the form of standardised and accepted procedures that are known to everyone. On the other hand, some stressed that it is still difficult to reach a consensus on certain questions, that not all situations can be anticipated and that the reasoning and the protocols would therefore not be applicable to all situations. As regards the involvement of the Ethics Committee in these discussions, emphasis was placed on the unique ethical aspects linked to the highly specialised care provided in NICUs, and on the difficulty of getting the members of an Ethics Committee to intervene within the very short time frame typically available in emergency situations. While palliative care in neonatology units has emerged as an important specific dimension in paediatrics [20], there is still some debate about the best possible practices to support the baby’s comfort while not prolonging its agony once a decision is made to withdraw life-sustaining interventions [21–23]. The results of our study reflect well the uncertainty inherent to this very complex area of care.

«Active» end-of-life is a difficult concept that may be interpreted differently by different people. It usually refers to the decision to administer drugs at above therapeutic doses with the intention of bringing about death. This is done in response to certain requests for euthanasia made by individuals who are considered to be able to make such a request. In the case of neonates, the question of euthanasia does not arise because the babies are unable to make such a request. «Active» end-of-life practices are in fact prohibited in Belgium. In the context of a palliative care pathway, however, doctors – faced with suffering that is considered intolerable on the part of the baby – may administer drugs to alleviate the baby’s pain, with the risk that these drugs will cause death. The intention here is therefore not to cause death but to alleviate suffering, with the concomitant risk that this will lead to death. Although the terminology varies across time and different contexts, this may be called continuous sedation until death [24].Our results show that continuous deep sedation until death is sometimes performed, after a deliberation process which involves the parents. In fact, the responses to the questionnaire suggest that there are situations where this is perceived as the more Human way of caring for the baby and their parents.

This raises the question of whether or not to standardise and/or legalise a practice which is currently at the margins of what is allowed in Belgium and in other countries. The fact that the desire for a law tended to be expressed by predominantly the youngest neonatologists in our study whereas the majority of the older neonatologists stated that they were uncertain whether or not they wanted a protocol or a law, seems to indicate a need for younger doctors to feel secure about the major decisions that they have to take. On the other hand, it probably reflects a greater awareness, gained through experience, of the individual and complex nature of each situation as well as a greater awareness of the risk resulting from excessively rigid wording that would limit the possibility of adapting decision-making to specific situations. Although some recommendations exist to guide the practitioners in countries such as the Netherlands where the so-called newborn “euthanasia” is allowed [10, 25], this issue certainly deserves further international and interdisciplinary debate, so as to help practitioners and parents come to terms with this extremely difficult issue when it arises.

## Conclusion

Our results reflect the uncertainty inherent in the complex and diverse situations that are encountered in this extreme area of clinical practice, and call for qualitative research and expert debates to further document and make recommendations for best practices regarding several “gray zones” of end-of-life care in neonatology, so that high quality palliative care may be granted to all neonates concerned with end-of-life decisions.

## Additional file

Additional file 1: Contains a printed version of the original online questionnaire in French. Authors are happy to help with the translation of the questionnaire on request. (PDF 103 kb)

## Abbreviations

NICU: Neonatal Intensive Care Unit
