# Supplementary material for: End-of-life decisions and practices for very preterm infants in the Wallonia-Brussels Federation of Belgium
Source: BMC Pediatr. 2018 Jun 26;18:206. doi: 10.1186/s12887-018-1168-x (PMC6020374; doi:10.1186/s12887-018-1168-x)
Supplement: Supplementary file 1 — Contains a printed version of the original online questionnaire in French. Authors are happy to help with the translation of the questionnaire on request. (PDF 103 kb) [file 12887_2018_1168_MOESM1_ESM.pdf]

Ce questionnaire a pour objet de réaliser un inventaire des principales pratiques des équipes médicales des services NIC (Neonatal Intensive Care) en Fédération Wallonie-Bruxelles dans les situations de naissance à la limite de la viabilité (<26 semaines). Il est adressé à tous les pédiatres travaillant dans un des 11 centres NIC.

Ce questionnaire comporte 68 questions et il faut **compter environ 60 minutes** pour le compléter.

Les questions vous interrogent sur **vos pratiques et votre vécu en matière de décisions de fin de vie aux limites de la viabilité**, à trois moments clés: la période anténatale, le moment de la naissance et la période post-natale. S'agissant d'un état des lieux sur les pratiques actuelles dans les différents centres, **les questions portent bien sur ce que vous faites effectivement au jour d'aujourd'hui et pas sur ce que vous pensez que vous-même ou votre centre devrait faire...** Si vous souhaitez vous exprimer sur des souhaits éventuels, vous pourrez le faire à la fin du questionnaire, dans un cadre réservé à cette effet.

Il n'y a pas de bonne ou de mauvaise réponse, et toutes les **données collectées sont totalement anonymes**.

Nous vous remercions de votre précieuse collaboration. Vos réponses seront très utiles pour décider des actions et recherches-actions futures qui seront soutenues par le Fonds Houtman dans les centres NIC.

Au nom du comité de pilotage du projet,

Isabelle Aujoulat

Coordinatrice de l'étude

Institut de Recherche Santé et Sociéte, UCL

E-mail : isabelle.aujoulat@uclouvain.be

Téléphone : 0487.66.41.49

Il y a 71 questions dans ce questionnaire

Données personnelles

1 [Genre]Vous êtes ?

Veuillez sélectionner **une seule** des propositions suivantes :

- ○ Un homme
- ○ Une femme

2 [Age]Quel est votre âge ?

Veuillez sélectionner **une seule** des propositions suivantes :

- ○ Moins de 35 ans
- ○ Entre 35 et 55 ans
- ○ Plus de 55 ans

3 [ANC-METIER]Depuis combien d'années exercez-vous le métier de pédiatre (nombre d'années écoulées depuis l'obtention de votre diplôme de médecin)?

Veuillez écrire votre réponse ici :

4 [ANC-NEONAT]Depuis combien d'années exercez-vous votre métier en néonatologie intensive ?

Veuillez écrire votre réponse ici :

5 [CENTRE]Quel est le centre qui vous emploie actuellement?

Choisissez **toutes** les réponses qui conviennent :

- ☐ Le CHR de La Citadelle
- ☐ Le CHC Clinique St-Vincent
- ☐ Le CHR de Namur
- ☐ Le CHU Tivoli
- ☐ Le CHU de Charleroi/Hôpital Civil
- ☐ Le Grand Hôpital de Charleroi
- ☐ Le CHU Saint-Pierre
- ☐ L'HUDERF
- ☐ L'Hôpital Erasme
- ☐ Les Cliniques universitaires Saint-Luc
- ☐ Le CHIREC, Clinique Edith Cavell
- ☐ Autre: ____________

L'information qui vous est demandée ici est importante pour nous permettre de vous restituer des résultats individualisés, concernant VOTRE centre. Ainsi, chaque participant recevra le rapport général concernant l'ensemble des 11 centres et une synthèse des résultats concernant son propre centre. Les données plus personnelles (âge, sexe, ancienneté dans le métier...) ne seront pas reprises dans les rapports individualisés mais présentées uniquement dans le rapport général pour décrire l'échantillon total des répondants. Les rapports individualisés ne seront transmis qu'aux centres concernés.

6 [CENTRE-AUTRE]Avez-vous, auparavant, travaillé dans un autre centre NIC (Neonatal Intensive Care) ?

Veuillez sélectionner **une seule** des propositions suivantes :

- ○ Oui, en Belgique
- ○ Oui, à l'étranger
- ○ Oui, en Belgique et à l'étranger
- ○ Non

7 [CENTRE-AUTRE2]Si oui, merci de préciser à quel moment de votre carrière ?

Choisissez **toutes** les réponses qui conviennent :

- ☐ pendant les 5 années de formation de pédiatrie
- ☐ pendant la formation de néonatologie
- ☐ après la reconnaissance de néonatologue

La prise en charge en PERIODE ANTENATALE

Les questions qui suivent vous interrogent sur votre expérience de prise en charge EN PERIODE ANTENATALE. Nous vous demandons de décrire votre expérience personnelle, le plus honnêtement possible, dans le centre dans lequel vous travaillez actuellement.

8 [DEC-ANT1]

Vous arrive-t-il de prendre des décisions anticipées (avant la naissance) de réanimation à la naissance ?

Veuillez sélectionner **une seule** des propositions suivantes :

- ○ Oui
- ○ Non

9 [DEC-ANT2]Qui est associé à la DISCUSSION des décisions anticipées de réanimation (ou non) à la naissance (consulté)?

Choisissez la réponse appropriée pour chaque élément :

	Toujours	Souvent	Parfois	Jamais	Sans avis
Gynécologue-obstétricien	○	○	○	○	○
Autre médecin spécialiste	○	○	○	○	○
Parents	○	○	○	○	○
Néonatologue	○	○	○	○	○
Médecin traitant	○	○	○	○	○
Psychologue	○	○	○	○	○
Sage-femme	○	○	○	○	○
Infirmière	○	○	○	○	○
Comité d'éthique	○	○	○	○	○
Autre	○	○	○	○	○

10 [DEC-ANTautre1]Si vous avez coché "autre" ou "autre médecin spécialiste", merci de préciser qui:

Veuillez écrire votre réponse ici :

11 [DEC-ANT3]Qui est associé à la DECISION en cas de décision anticipée de réanimation (ou non) à la naissance?

Choisissez la réponse appropriée pour chaque élément :

	Toujours	Souvent	Parfois	Jamais	Sans avis
Gynécologue-obstétricien	○	○	○	○	○
Autre médecin spécialiste	○	○	○	○	○
Parents	○	○	○	○	○
Néonatologue	○	○	○	○	○
Médecin traitant	○	○	○	○	○
Psychologue	○	○	○	○	○
Sage-femme	○	○	○	○	○
Infirmière	○	○	○	○	○
Comité d'éthique	○	○	○	○	○
Autre	○	○	○	○	○

12 [DEC-ANTautre2]Si vous avez coché "autre" ou "autre médecin spécialiste" à la question précédente, merci de préciser qui:

Veuillez écrire votre réponse ici :

13 [POIDS-DEC]Qui a le plus de poids dans la décision finale de réanimation (ou non) à la naissance ? (Veuillez classer par ordre d'importance de 1 à 4, 1 étant le plus important et 4 le moins important)

Veuillez saisir une nombre compris entre 1 et 4 pour chaque élément :

	Les parents	L'équipe obstétricale	Les néonatologues	Autre ou ça dépend
AVANT 26 semaines				
APRES 26 semaines				

Classer par ordre d'importance de 1 à 4, 1 étant le plus important et 4 le moins important.

14 [POIDS-DEC2]Si vous avez choisi "autre ou ça dépend" à la question précédente, pouvez vous commenter ?

Veuillez écrire votre réponse ici :

15 [PROTOC]

Existe-t-il un protocole écrit ou une procédure standardisée pour l'anticipation anténatale d'une réanimation (ou non) à la naissance ?

Veuillez sélectionner **une seule** des propositions suivantes :

- Oui
- Non

16 [TRANS-mic]

Dans votre centre, à partir de quel âge gestationnel l'équipe du MIC (Maternal Intensive Care) accepte-t-elle un transfert maternel in utero en vue d'une possible réanimation du bébé ?

Veuillez sélectionner **une seule** des propositions suivantes :

- moins de 22 semaines
- 22 semaines
- 23 semaines
- 24 semaines
- 25 semaines
- 26 semaines et plus

17 [TRANS-Nic]

Dans votre centre, à partir de quel âge gestationnel l'équipe des néonatologues accepte-t-elle de prendre le bébé en réanimation ?

Veuillez sélectionner **une seule** des propositions suivantes :

- moins de 22 semaines
- 22 semaines
- 23 semaines
- 24 semaines
- 25 semaines
- 26 semaines et plus

18 [MAT-CORT]

Dans votre centre, à partir de quel âge gestationnel commencez-vous une maturation par corticoïdes ?

Veuillez sélectionner **une seule** des propositions suivantes :

- moins de 22 semaines
- 22 semaines
- 23 semaines
- entre 23 et 24 semaines
- 24 semaines
- 25 semaines
- 26 semaines et plus

19 [ANAMN]

Dans votre centre, quels critères anamnestiques sont pris en compte pour prendre la décision de réanimer ou non à la naissance?

Choisissez la réponse appropriée pour chaque élément :

	Toujours	Souvent	Parfois	Jamais	Je ne sais pas
Singleton / multiple	○	○	○	○	○
Sexe (fille / garçon)	○	○	○	○	○
Phénotype (couleur de peau noire/blanche)	○	○	○	○	○
Poids à la naissance ou retard de croissance intrautérin	○	○	○	○	○
Milieu socioéconomique favorable ou défavorable	○	○	○	○	○
Toxicomanie maternelle	○	○	○	○	○
Souffrance fœtale aigüe	○	○	○	○	○
Présence de malformation significative	○	○	○	○	○
Age maternel	○	○	○	○	○
Passé obstétrical	○	○	○	○	○
Chorioamniotite, autre infection	○	○	○	○	○
Conviction philosophique ou religieuse des parents	○	○	○	○	○
Autre	○	○	○	○	○

20 [CRIT-autre]Pouvez-vous indiquer quel(s) autre(s) critère(s) sont éventuellement pris en compte?

Veuillez écrire votre réponse ici :

21 [FOMAL-DEC]Comment est formalisée et consignée la décision finale de réanimation ou non ?

Choisissez la réponse appropriée pour chaque élément :

	Toujours	Souvent	Parfois	Jamais	Je ne sais pas
Les parents sont invités à signer la décision de réanimation ou non	○	○	○	○	○
La décision est inscrite dans le dossier maternel	○	○	○	○	○
Les détails de la discussion qui précède la décision sont inscrits dans le dossier maternel	○	○	○	○	○

La prise en charge A LA NAISSANCE

Les questions qui suivent vous interrogent sur votre expérience de prise en charge AU MOMENT DE LA NAISSANCE. Nous vous demandons de décrire votre expérience personnelle, le plus honnêtement possible, dans le centre dans lequel vous travaillez actuellement.

22 [NON-RESPECT DEC]En cas de décision anténatale de réanimation à la naissance, arrive-t-il qu'un médecin de garde n'applique pas la décision qui avait été prise en période anténatale ?

Veuillez sélectionner **une seule** des propositions suivantes :

- ○ Oui
- ○ Non

23 [CRIT-MODIF DEC]Une décision anténatale de réanimation (ou non) peut-elle être remise en cause par:

Choisissez la réponse appropriée pour chaque élément :

	OUI	NON	Je ne sais pas
L'évaluation clinique de la maturité de l'enfant	○	○	○
La vitalité du bébé à la naissance	○	○	○
La présence de malformation(s) non décelée(s) pendant la grossesse	○	○	○
Autre	○	○	○

24 [CRIT-MODIF2]Si vous avez répondu "autre" à la question précédente, merci de préciser :

Veuillez écrire votre réponse ici :

25 [ABSENCE-DEC]En l'absence de décision anténatale de réanimation (ou non) à la naissance, à partir de quel âge gestationnel appelle-t-on le néonatologue ?

Choisissez la réponse appropriée pour chaque élément :

	Néonatologue senior en premier recours	Néonatologue junior (assistant en pédiatrie ou médecin en formation) en premier recours	L'un ou l'autre suivant disponibilité	Ni l'un ni l'autre	Je ne sais pas
moins de 22 semaines	○	○	○	○	○
22 semaines	○	○	○	○	○
23 semaines	○	○	○	○	○
24 semaines	○	○	○	○	○
25 semaines	○	○	○	○	○
26 semaines et plus	○	○	○	○	○

26 [QUI-DEC]

Qui est associé à la décision de réanimation (ou non) en cas de naissance urgente sans décision anticipée ?

Choisissez la réponse appropriée pour chaque élément :

	Toujours	Souvent	Parfois	Jamais	Je ne sais pas
Gynécologue-obstétricien	○	○	○	○	○
Autre médecin spécialiste	○	○	○	○	○
Parents	○	○	○	○	○
Néonatologue	○	○	○	○	○
Médecin traitant	○	○	○	○	○
Psychologue	○	○	○	○	○
Sage-femme	○	○	○	○	○
Infirmière	○	○	○	○	○
Comité d'éthique	○	○	○	○	○
Autre	○	○	○	○	○

27 [QUI-DECautre]Si vous avez indiqué "autre" ou "un autre médecin spécialiste" à la question précédente, merci de préciser:

Veuillez écrire votre réponse ici :

28 [ANAMN]

Quels critères anamnestiques sont pris en compte pour prendre la décision de réanimer ou non en cas de naissance urgente ?

Choisissez la réponse appropriée pour chaque élément :

	Toujours	Souvent	Parfois	Jamais	Je ne sais pas
Singleton / multiple	○	○	○	○	○
Sexe (fille / garçon)	○	○	○	○	○
Phénotype (couleur de peau noire/blanche)	○	○	○	○	○
Poids à la naissance ou retard de croissance intrautérin	○	○	○	○	○
Milieu socioéconomique favorable ou défavorable	○	○	○	○	○
Toxicomanie maternelle	○	○	○	○	○
Souffrance fœtale aigüe	○	○	○	○	○
Présence de malformation significative	○	○	○	○	○
Age maternel	○	○	○	○	○
Passé obstétrical	○	○	○	○	○
Chorioamniotite, autre infection	○	○	○	○	○
Conviction philosophique ou religieuse des parents	○	○	○	○	○

29 [CRIT-autre]Si d'autres critères interviennent dans la décision de réanimer (ou non) en cas de naissance urgente, merci de préciser lesquels:

Veuillez écrire votre réponse ici :

30 [REA-ATTENTE]

Vous arrive-t-il de réaliser une réanimation d’attente, le temps de clarifier la situation (attitude des parents, bébé très dynamique, âge gestationnel incertain, anamnèse lacunaire)?

Veuillez sélectionner **une seule** des propositions suivantes :

○ Oui

○ Non

31 [PALLIATIF]En cas de décision de non-réanimation, qui se charge des soins palliatifs?

Choisissez la réponse appropriée pour chaque élément :

	Sage-femme	Gynécologue-obstétricien	Néonatologue senior	Néonatologue junior	Infirmière	Autre ou ça dépend
moins de 22 semaines	○	○	○	○	○	○
22 semaines	○	○	○	○	○	○
23 semaines	○	○	○	○	○	○
24 semaines	○	○	○	○	○	○
25 semaines	○	○	○	○	○	○
26 semaines et plus	○	○	○	○	○	○

32 [PALLIA-autre]Si vous avez indiqué "autre ou ça dépend" à la question précédente, merci de préciser :

Veuillez écrire votre réponse ici :

33 [LIEU-PALLIAT]Où se passent les soins palliatifs ?

Choisissez la réponse appropriée pour chaque élément :

	OUI	NON	Autre ou ça dépend
Dans la salle d'accouchement	○	○	○
Dans le service néonatal	○	○	○

34 [LIEU-autre]Si vous avez indiqué "autre ou ça dépend", merci de préciser:

Veuillez écrire votre réponse ici :

35 [PROTOC]Avez-vous un protocole écrit ou une procédure standardisée pour assurer le confort du bébé dans le cadre du suivi en soins palliatifs ?

Veuillez choisir toutes les réponses qui conviennent et laissez un commentaire :

- ☐ Oui, un protocole médicamenteux
- ☐ Oui, un autre type de protocole. Merci de préciser lequel :
- ☐ Non
- ☐ Je ne sais pas

La prise en charge en PERIODE POSTNATALE

Les questions qui suivent vous interrogent sur votre expérience de prise en charge APRES LA NAISSANCE. Nous vous demandons de décrire votre expérience personnelle, le plus honnêtement possible, dans le centre dans lequel vous travaillez actuellement.

36 [DEC-VIAB]

S'il y a constat que la poursuite de soins est déraisonnable, le processus décisionnel de passage en soins palliatifs est-il comparable si l'enfant est au seuil de la viabilité (< 26 semaines) ou s'il est plus âgé (> 26 semaines) ?

Veuillez sélectionner **une seule** des propositions suivantes :

- ○ OUI
- ○ NON
- ○ Je ne sais pas

37 [DEC-POURS]

Qui est inclus dans le processus décisionnel relatif à la poursuite ou l'arrêt des soins ?

Choisissez la réponse appropriée pour chaque élément :

	Toujours	Souvent	Parfois	Jamais	Sans avis
Néonatologue	○	○	○	○	○
Gynécologue-obstétricien	○	○	○	○	○
Autre médecin spécialiste	○	○	○	○	○
Parents	○	○	○	○	○
Médecin traitant	○	○	○	○	○
Psychologue	○	○	○	○	○
Sage-femme	○	○	○	○	○
Infirmière	○	○	○	○	○
Comité d'éthique	○	○	○	○	○
Autre	○	○	○	○	○

38 [DEC-autre]Si vous avez indiqué "autre" ou "autre médecin spécialiste" à la question précédente, merci de préciser qui :

Veuillez écrire votre réponse ici :

39 [DEC-ARRET]En fonction de l'âge gestationnel, qui prend formellement la décision d'arrêter les soins ?

Choisissez la réponse appropriée pour chaque élément :

	AVANT 26 semaines ?					APRES 26 semaines ?				
	Toujours	Souvent	Parfois	Jamais	Sans avis	Toujours	Souvent	Parfois	Jamais	Sans avis
Parents	○	○	○	○	○	○	○	○	○	○
Néonatologue	○	○	○	○	○	○	○	○	○	○
Gynécologue-obstétricien	○	○	○	○	○	○	○	○	○	○
Autre	○	○	○	○	○	○	○	○	○	○

40 [DESAC]En cas de désaccord, quelle est la procédure de prise de décision ?

Veuillez écrire votre(vos) réponse(s) ici :

AVANT 26 semaines

APRES 26 semaines

41 [EXP]Avez-vous une équipe d'experts extérieurs à votre service pour vous aider à prendre la décision de réanimation ou non :

Veuillez sélectionner **une seule** des propositions suivantes :

- Oui
- Non

42 [OUI-PREC]Si oui, merci de préciser:

Veuillez écrire votre réponse ici :

43 [EXP2]Avez-vous une équipe d'experts extérieurs à votre service pour vous aider à pratiquer les soins palliatifs?

Veuillez sélectionner **une seule** des propositions suivantes :

- Oui
- Non

44 [OUI-PREC2]Si oui, merci de préciser :

Veuillez écrire votre réponse ici :

45 [CRIT-Arrêt]La décision d’arrêt des soins curatifs est-elle influencée par?

Choisissez la réponse appropriée pour chaque élément :

	Toujours	Presque toujours	Souvent	Parfois	Exceptionnellement	Jamais
La qualité de vie ultérieure supposée du bébé	○	○	○	○	○	○
La qualité de vie ultérieure supposée de la famille	○	○	○	○	○	○
Le pronostic vital à +/- brève échéance engagé	○	○	○	○	○	○
Le pronostic morbide ultérieur	○	○	○	○	○	○
Le vécu parental au moment de la naissance	○	○	○	○	○	○
Autre	○	○	○	○	○	○

46 [CRIT-autre]Si vous avez indiqué "autre" à la question précédente, merci de précisier :

Veuillez écrire votre réponse ici :

47 [PROJ-THER]

Construisez-vous pour chaque enfant à la limite de viabilité un projet thérapeutique? Merci de préciser en fonction de l'âge gestationnel.

Choisissez la réponse appropriée pour chaque élément :

	AVANT 26 semaines				APRES 26 semaines			
	OUI	NON	Ca dépend	Je ne sais pas	OUI	NON	Ca dépend	Je ne sais pas
En cas de soins curatifs	○	○	○	○	○	○	○	○
En cas de soins palliatifs	○	○	○	○	○	○	○	○

48 [EVOL]Si un projet thérapeutique existe, est-il évolutif ?

Veuillez sélectionner **une seule** des propositions suivantes :

- OUI
- NON
- Ca dépend
- Je ne sais pas

Faites le commentaire de votre choix ici :

49 [DOSS MED]Si un projet thérapeutique existe, est-il inscrit dans le dossier médical ?

Veuillez sélectionner **une seule** des propositions suivantes :

- Toujours
- Souvent
- Parfois
- Jamais
- Je ne sais pas

Faites le commentaire de votre choix ici :

50 [DOSS INF]Si un projet thérapeutique existe, est-il inscrit dans le dossier infirmier ?

Veuillez sélectionner **une seule** des propositions suivantes :

- Toujours
- Souvent
- Parfois
- Jamais
- Je ne sais pas

Faites le commentaire de votre choix ici :

51 [AVIS PARENTS]Vous arrive-t-il de démarrer un projet thérapeutique palliatif...

Choisissez la réponse appropriée pour chaque élément :

	Oui	Incertain	Non
...sans en informer les parents	○	○	○
...contre l'avis des parents	○	○	○

52 [FIn ACTIVE]Dans le cadre d'un projet thérapeutique palliatif, vous arrive-t-il de pratiquer une fin de vie active (utilisation de médicaments d'analgésie et/ou de sédation à des doses supra thérapeutiques) ?

Veuillez sélectionner **une seule** des propositions suivantes :

- ○ OUI
- ○ NON
- ○ Je ne sais pas

Faites le commentaire de votre choix ici :

53 [INFO PARENTS]En cas de décision de pratiquer une fin de vie active, en informez-vous les parents?

Veuillez sélectionner **une seule** des propositions suivantes :

- ○ Toujours
- ○ Souvent
- ○ Parfois
- ○ Jamais
- ○ Je ne pratique jamais de fin de vie active
- ○ Sans avis

54 [MOTIF]Lorsque vous pratiquez une fin de vie active, les motifs de la décision sont :

Choisissez la réponse appropriée pour chaque élément :

	Toujours	Souvent	Parfois	Exceptionnellement	Jamais	Je ne sais pas
Eviter une qualité de vie future médiocre (conviction qu'il y aura un handicap majeur)	○	○	○	○	○	○
Soulager des souffrances chez le bébé, jugées inutiles	○	○	○	○	○	○
Répondre aux demandes (pressions) parentales	○	○	○	○	○	○
Répondre aux demandes (pressions) de l'équipe soignante	○	○	○	○	○	○
Autre	○	○	○	○	○	○

55 [MOTIF-autre]Si vous avez indiqué "autre" à la question précédente, merci de préciser :

Veuillez écrire votre réponse ici :

56 [DECES]En cas de décès, proposez-vous :

Choisissez la réponse appropriée pour chaque élément :

	Toujours	Souvent	Parfois	Jamais	Je ne sais pas
Une autopsie ?	○	○	○	○	○
Une IRM ?	○	○	○	○	○
Les 2 ?	○	○	○	○	○

57 [TEMPS]Vous arrive-t-il de postposer le moment du décès, afin de laisser du temps...

Choisissez la réponse appropriée pour chaque élément :

	Oui	Incertain	Non
...au cheminement des parents	○	○	○
...au cheminement de l'équipe	○	○	○

58 [RITUELS]Proposez-vous des rituels standardisés après le décès du bébé?

Veuillez sélectionner **une seule** des propositions suivantes :

○ Oui

○ Non

59 [RITUELS1]Si vous proposez des rituels après le décès, qui se charge préférentiellement de ces rituels ?

Choisissez la réponse appropriée pour chaque élément :

	Infirmière des soins	Infirmière sociale	Sage-femme	Néonatologue	Psychologue	Ministre du culte	Bénévole de l'aumônerie	Membre d'une association de parents	Autre	Nous ne proposons pas ce rituel
Religieux	○	○	○	○	○	○	○	○	○	○
Accueil des membres élargis de la famille	○	○	○	○	○	○	○	○	○	○
Objet souvenir (photo, empreinte, cheveux)	○	○	○	○	○	○	○	○	○	○
Autre	○	○	○	○	○	○	○	○	○	○

60 [RITUELautre]Si vous pratiquez d'autres rituels ou que d'autres personnes sont impliquées, merci de préciser:

Veuillez écrire votre réponse ici :

61 [ACC]Proposez-vous un accompagnement des parents endeuillés ?

Choisissez la réponse appropriée pour chaque élément :

	Toujours	Souvent	Parfois	Jamais	Sans avis
En cas de décès du bébé à la naissance SANS REANIMATION	○	○	○	○	○
En cas de décès du bébé APRES REANIMATION	○	○	○	○	○

62 [ACC2]Si vous proposez un accompagnement des parents endeuillés, quelles formes d'accompagnement proposez-vous et qui en prend l'initiative ?

Choisissez la réponse appropriée pour chaque élément :

	ACCOMPAGNEMENT AU MOMENT DU DECES					ACCOMPAGNEMENT A DISTANCE DU DECES				
	Toujours proposé	Proposé si les parents en font la demande	Proposé si nous percevons que les parents sont en difficulté	L'initiative peut venir des parents ou de notre équipe	Jamais proposé	Toujours proposé	Proposé si les parents en font la demande	Proposé si nous percevons que les parents sont en difficulté	L'initiative peut venir des parents ou de notre équipe	Jamais proposé
Groupe de parole	○	○	○	○	○	○	○	○	○	○
Rencontre avec un(e) psychologue au sein de l'hôpital	○	○	○	○	○	○	○	○	○	○
Orientation vers une association	○	○	○	○	○	○	○	○	○	○
Contact avec des parents ressources	○	○	○	○	○	○	○	○	○	○
Autre	○	○	○	○	○	○	○	○	○	○

63 [ACC-autre]Si vous avez coché "autre" à la question précédente, pouvez-vous préciser:

Veuillez écrire votre réponse ici :

64 [ACC3]Lorsque les parents sont revus à distance du décès, l'initiative provient de :

Veuillez choisir toutes les réponses qui conviennent et laissez un commentaire :

- ☐ L'équipe soignante
- ☐ Les parents
- ☐ Ca dépend

Aspect légal

Cette partie vous interroge sur ce que vous pensez personnellement en lien avec les aspects formels et juridiques liés aux décisions de fin de vie en néonatologie.

65 [PEUR]Lorsque vous prenez une décision, vous arrive-t-il de craindre d'éventuelles actions médico-légales et/ou poursuites judiciaires ?

Choisissez la réponse appropriée pour chaque élément :

	CRAINTES POUR VOUS-MEME					CRAINTES POUR VOTRE CENTRE				
	Toujours	Souvent	Parfois	Exceptionnellement	Jamais	Toujours	Souvent	Parfois	Exceptionnellement	Jamais
En cas de décision de non-réanimation	○	○	○	○	○	○	○	○	○	○
En cas de décision de soins palliatifs (arrêt des soins curatifs)	○	○	○	○	○	○	○	○	○	○
En cas de décision de pratique de fin de vie active	○	○	○	○	○	○	○	○	○	○

66 [PEUR-qui]S'il vous arrive de craindre d'éventuelles actions médico-légales et/ou poursuites judiciaires, qui peut être à la source de vos craintes?

Choisissez la réponse appropriée pour chaque élément :

	Oui, cette craint a déjà existé	Oui, cette crainte pourrait exister	Non
Les parents	○	○	○
Des membres du personnel	○	○	○
Autre ou ça dépend	○	○	○

67 [PEUR-autre]Si vous avez indiqué "autre ou ça dépend" à la question précédente, pouvez-vous préciser :

Veuillez écrire votre réponse ici :

68 [TXT]Souhaitez-vous que les conduites à tenir en cas de fin de vie active dans les situations de grande prématurité soient encadrées...

Choisissez la réponse appropriée pour chaque élément :

	Oui	Incertain	Non
par un protocole	○	○	○
par un texte légal	○	○	○

69 [TXT2]Souhaitez-vous que les pratiques de fin de vie actives dans les situations de grande prématurité soient autorisées...

Choisissez la réponse appropriée pour chaque élément :

	Oui	Incertain	Non
par un protocole	○	○	○
par un texte légal	○	○	○

70 [COMM]Vous pouvez commenter vos réponses ici si vous le souhaitez :

Veuillez écrire votre réponse ici :

Commentaire final

71 [COMM FIN]Vous arrivez à la fin du questionnaire. Après avoir cliqué sur le bouton "envoyer", il ne vous sera plus possible de revenir en arrière. Si vous souhaitez laisser un commentaire ou poser une question, vous pouvez le faire ici :

Veuillez écrire votre réponse ici :

Merci d'avoir répondu à ce questionnaire.

Pour toute question ou préoccupation, n'hésitez pas à prendre contact avec moi ou avec le responsable de votre centre.

Isabelle Aujoulat

Coordinatrice de l'étude

Institut de Recherche Santé et Sociéte, UCL

E-mail : isabelle.aujoulat@uclouvain.be

Téléphone : 0487.66.41.49

01.01.1970 – 01:00

Envoyer votre questionnaire.
Merci d'avoir complété ce questionnaire.
